# Transcriptomic profiling of taxol-resistant ovarian cancer cells identifies FKBP5 and the androgen receptor as critical markers of chemotherapeutic response

**DOI:** 10.18632/oncotarget.2654

**Published:** 2014-11-17

**Authors:** Nian-Kang Sun, Shang-Lang Huang, Pu-Yuan Chang, Hsing-Pang Lu, Chuck C.-K. Chao

**Affiliations:** ^1^ Department of Biochemistry and Molecular Biology, College of Medicine, Chang Gung University, Taoyuan 333, Taiwan, Republic of China; ^2^ Division of Biomedical Sciences, Chang Gung University of Science and Technology, Taoyuan 333, Taiwan, Republic of China; ^3^ Graduate Institute of Biomedical Sciences, College of Medicine, Chang Gung University, Taoyuan 333, Taiwan, Republic of China

## Abstract

Taxol is a mitotoxin widely used to treat human cancers, including of the breast and ovary. However, taxol resistance (txr) limits treatment efficacy in human patients. To study chemoresistance in ovarian cancer, we established txr ovarian carcinoma cells derived from the SKOV3 cell lineage. The cells obtained were cross-resistant to other mitotoxins such as vincristine while they showed no resistance to the genotoxin cisplatin. Transcriptomic analysis identified 112 highly up-regulated genes in txr cells. Surprisingly, FK506-binding protein 5 (FKBP5) was transiently up-regulated 100-fold in txr cells but showed decreased expression in prolonged culture. Silencing of FKBP5 sensitized txr cells to taxol, whereas ectopic expression of FKBP5 increased resistance to the drug. Modulation of FKBP5 expression produced similar effects in response to vincristine but not to cisplatin. We observed that a panel of newly identified txr genes was trancriptionally regulated by FKBP5 and silencing of these genes sensitized cells to taxol. Notably, immunoprecipitation experiments revealed that FKBP5 forms a protein complex with the androgen receptor (AR), and this complex regulates the transcriptional activity of both proteins. Furthermore, we found that the Akt kinase pathway is regulated by FKBP5. These results indicate that the FKBP5/AR complex may affect cancer cell sensitivity to taxol by regulating expression of txr genes. Our findings suggest that mitotoxin-based treatment against ovarian cancer should be avoided when the Akt/FKBP5/AR axis is activated.

## INTRODUCTION

The taxanes paclitaxel (taxol) and docetaxel are microtubule-stabilizing agents that function primarily by interfering with spindle microtubule dynamics, ultimately causing cell cycle arrest and apoptosis. These agents have become widely recognized as active chemotherapeutic agents for the treatment of various human cancers. However, their therapeutic efficacy is limited by inherent or acquired resistance [1, 2]. Membrane transporters of the ATP-binding cassette (ABC) and solute carrier (SLC) families play a major role in these phenomena. Probably the most important ABC protein in this context is glycoprotein P (P-gp), which is encoded by the abcb1 gene (multidrug resistance protein 1, or MDR1) [3]. This protein is a drug efflux pump that can actively remove nearly 20 different drugs from the cell. It is expected that at least 10 additional ABC proteins are involved in drug resistance [4]. Structural advances in this field have provided a framework to decipher the kinetic and molecular mechanisms by which ABC transporters couple ATP hydrolysis to substrate translocation [5]. Another group of membrane transporters involved in drug resistance is the SLC transporters, which function mainly as influx transporters [6]; these transporters are often downregulated in chemoresistant cells [7–9]. Despite recent advances in this field, no valid biomarkers exist to predict resistance to taxanes in breast cancer [1]. Overexpression of MDR-1/P-gp and altered expression of microtubule-associated proteins (MAPs), including tau, stathmin, and MAP4, may help identify the patients who are at risk of recurrence and the ones most likely to benefit from taxane treatment [2]. Gene set enrichment analysis (GSEA) is a statistical methodology for determining whether a given gene set is significantly associated with a phenotype of interest [10, 11]. GSEA has been successfully used to identify metabolic pathways altered in many diseases, including to identify that activation of the PI3K/Akt pathway is associated with incomplete metabolic response in cervical cancer [12].

PI3K is activated by growth factor signaling through both Ras and receptor kinase signaling. One of the early events in Akt activation is the recruitment of PIP3 to the cellular membrane. Akt becomes fully activated by phosphorylation at two sites, S473 and T308. In contrast, the phosphatases in the PH domain and leucine-rich repeat protein phosphatases (PHLPP) family have been shown to directly dephosphorylate and therefore inactivate distinct Akt isoforms. FKBP5 functions as a scaffolding protein that brings PHLLP closer to the Akt S473 site and facilitate the dephosphorylation of S473, which in turn downregulates Akt signaling [13]. The protein kinase Akt regulates cellular survival [14] and metabolism by binding and regulating many downstream effectors. Furthermore, Akt is frequently activated in human cancers and has been implicated in resistance to chemotherapy.

FK506 binding protein 5 (FKBP5) belongs to a family of immunophilins that exhibit peptidylprolyl *cis/trans* isomerase (PPIase) activity [15, 16]. FKBP5, a target for drugs such as rapamycin and tacrolimus (FK506), binds proteins such as Akt and the progesterone receptor (PR) at FKBP-type domains. FKBP5 also binds the androgen receptor (AR), glucocorticoid receptor (GR), phosphatase PHLPP, and chaperone Hsp90 through tetratricopeptide repeat (TPR) domains. FKBP5 is involved in several signaling pathways, including hormone signaling, irradiation-induced NF-κB activation, and chemotherapy-induced Akt-PHLPP pathways, exerting important roles in cancer development and chemoresistance [17]. Although FKBP5 shares many characteristics with other FK506 binding proteins (FKBPs), it also has unique features, such as regulating important signaling pathways (e.g., Akt) [13]. FKBP5 is highly expressed in multiple tissues. Previous studies showed that upregulation of FKBP5 is associated with drug resistance in various cancers (including breast, prostate, myeloma, acute lymphoblastic leukemia, melanoma) following treatment with antineoplastic agents (FK506, rapamycin, dexamethasone, irradiation) [18–23]. These studies involved NF-κB or hormone signaling. In contrast, Wang and colleagues used genome-wide screening to demonstrate that FKBP5 levels are inversely associated with response to two cytidine analogues, gemcitabine and cytosine arabinoside [24]. Furthermore, downregulation of FKBP5 desensitized pancreatic and breast cancer cell lines to several classes of chemotherapeutic agents, including not only cytidine analogues but also taxanes, irinotecan, and etoposide [24, 25]. These findings can be explained by the regulatory mechanisms affecting Akt kinase activity which are also involved in regulating chemosensitivity [26].

By influencing Akt phosphorylation, FKBP5 cellular levels may affect sensitivity to chemotherapy. However, the actions of FKBP5 on NF-κB activation or hormone induction could not explain the increased level of chemoresistance in cells showing reduced FKBP5 expression, suggesting the existence of other mechanisms by which FKBP5 may regulate cell survival. FKBP5 expression can be induced by activation of AR, GR, and PR [27]. Both overexpression and downregulation of FKBP5 have been observed in human cancers. FKBP5 has been shown to either promote or suppress tumor growth through its regulation of different signaling pathways in specific tissue environments [17].

Considering that taxanes are important chemotherapeutic agents for the treatment of cancers, we are trying to determine the mechanism of taxol resistance (txr) in order to optimize the use of these drugs during cancer treatment. Using a microarray analysis, we searched for new candidate genes in SKOV3-derived txr cell lines in order to identify txr biomarkers. Among the txr genes identified, FKBP5 is the most notable since it is highly upregulated early during development of txr, but show reduced expression in prolonged culture. To our knowledge, this study is the first report revealing that FKBP5 plays a role in survival signaling and taxol response in ovarian cancer.

## RESULTS

### FKBP5 modulates cell response to mitotoxins but not to genotoxins

**Figure 1:**
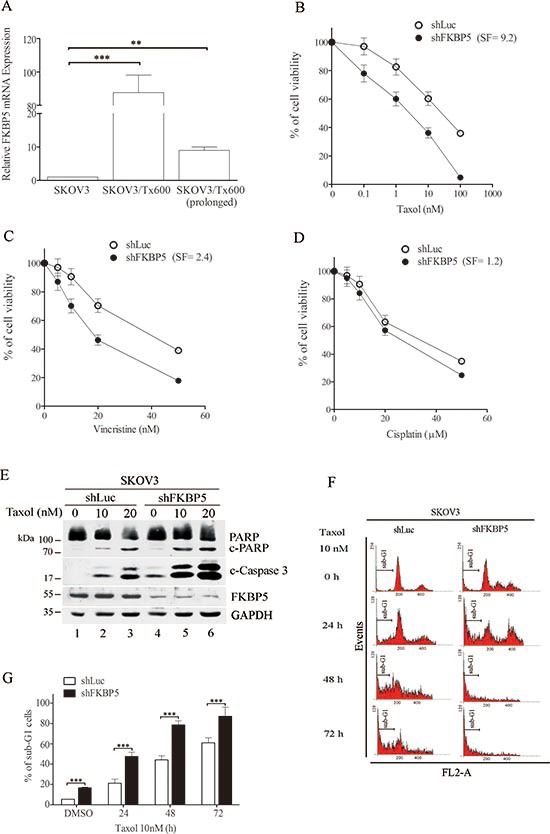
Silencing FKBP5 sensitizes ovarian cancer cells to taxol **(A)** Upregulation of FKBP5 in txr cells. **(B)** Cell sensitization to taxol following silencing of FKBP5. **(C)** Cell sensitization to vincristine following silencing of FKBP5. **(D)** Lack of cell sensitization to cisplatin by FKBP5 silencing. Cell viability was assessed by the MTT assay. **(E)** Potentiation of caspase activation by silencing FKBP5. Cleavage of caspase-3 and PARP is indicated. **(F)** Representative sub-G1 cell profiling following FKBP5 silencing. **(G)** Potentiation of sub-G1 cells by FKBP5 silencing. The results are expressed as mean values ± SD for experiments performed in triplicate. Statistical significance: *P < 0.05; **P < 0.01; ***P < 0.001.

Transcriptomic profiling revealed that FKBP5 mRNA was highly up-regulated in txr cells (the DNA microarray data were deposited into the GEO database; GSE60335). Surprisingly, up-regulation of FKBP5 was nearly lost after prolonged culture (12 months; GSE58878 containing the subseries GSE58840 and GSE58877). Accordingly, FKBP5 expression was dramatically reduced in prolonged culture of txr cells as measured by qPCR (Fig. 1A). We examined the kinetic change of FKBP5 gene expression during early response to taxol (challenged every other day). The mRNA level measured by qPCR was slightly induced after taxol treatment (1 nM), reaching a peak at day 1 and declining thereafter to non-induced level. Although a second challenge with the same concentration of taxol did not cause immediate increase of FKBP5's slope or peak, a third challenge led to a large increase of FKBP5, followed by a moderate decline (Fig. S1). These results indicate that the FKBP5 gene is inducible and its expression increases at the early stage of txr development, following taxol treatment. However, the FKBP5 gene is less responsive to the drug in late culture during selection with increasing concentration of taxol. The level of FKBP5 was dramatically reduced in txr cells compared to early cultures, but it still increased 10 fold compared to non-treated cells.

To assess the role of FKBP5, we silenced this gene using short-hairpin RNA (shRNA). FKBP5 silencing (shFKBP5) significantly sensitized txr cells to taxol (Fig. 1B). We examined the effects of FKBP5 silencing in response to others chemotherapeutic drugs. While FKBP5 silencing sensitized txr cells to taxol by 9.2-fold, a 2.4-fold sensitization effect was noted for vincristine (Fig. 1C). Notably, silencing of FKBP5 did not affect cell response to the genotoxin cisplatin (Fig. 1D), suggesting that taxol-regulated genes such as FKBP5 may be regulated differently by DNA-damaging agents.

To ensure that the sensitization effect was associated with apoptosis, we examined caspase-3 activation using Western blotting. In control SKOV3 cells expressing shLuc, taxol induced cleavage and activation of caspase-3 and its substrate, poly-ADP ribose polymerase (PARP). Caspase-3 activation increased in taxol-treated cells following silencing of FKBP5 (Fig. 1E). Enhanced apoptosis was also confirmed by the significant increase of sub-G1 cells following FKBP5 silencing (see Fig. 1F for representative flow cytometry diagrams and Fig. 1G for the corresponding quantitative and statistical analysis; *P* < 0.001).

**Figure 2:**
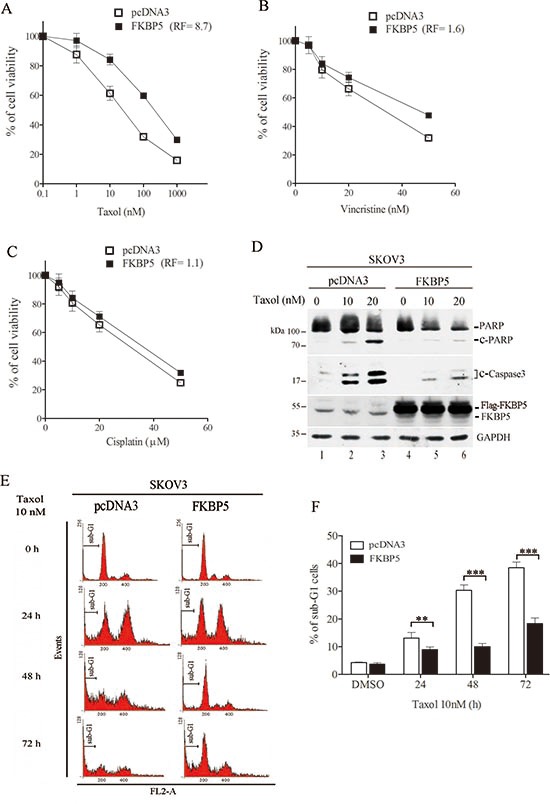
Ectopic expression of FKBP5 protects cells against taxol toxicity **(A)** Cell resistance to taxol induced by FKBP5 overexpression. **(B)** Cell resistance to vincristine induced by FKBP5 overexpression. **(C)** Lack of modification on cell response to cisplatin in cells overexpressing FKBP5. **(D)** Inhibition of caspase activation by FKBP5 overexpression. (E) Representative sub-G1 cell profiling following FKBP5 overexpression. **(F)** Inhibition of sub-G1 cells in FKBP5 oxerexpressing cells. Symbols are same as for Fig. 1

In contrast, ectopic expression of FKBP5 induced resistance to taxol by 8.7 fold (Fig. 2A). We observed that FKBP5 overexpression increased resistance to vincristine by 1.8 fold (Fig. 2B). However, FKBP5 overexpression did not significantly alter cellular response to cisplatin (Fig. 2C). Ectopic expression of FKBP5 significantly reduced caspase activation and sub-G1 cell accumulation induced by taxol in SKOV3 cells (Fig. 2D-F). Taken together, these results show that FKBP5 modulates cell response to mitotoxins.

### FKBP5 expression reduces activation of pro-apoptotic mitochondrial pathways

**Figure 3:**
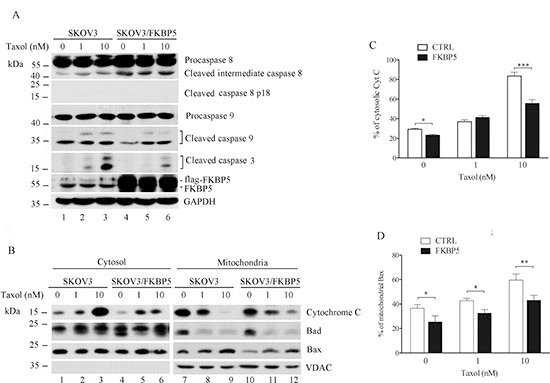
Reduction of mitochondrial apoptotic pathway by ectopic expression of FKBP5 in ovarian cancer cells **(A)** Reduction of caspase-9 activation by FKBP5 overexpression. Taxol-induced caspase cleavage is indicated. Cells were transfected with Flag-FKBP5. **(B)** Modification of apoptotic markers in cytoplasmic and mitochondrial fractions following FKBP5 overexpression. VDAC was used as a marker for fractionation. **(C)** Reduction of cytosolic cytochrome c by FKBP5 overexpression. **(D)** Reduction of mitochondrial Bax following FKBP5 overexpression. The results of (C) and (D), derived from (B), are expressed as mean values ± SD for experiments performed in triplicate. Statistical significance: *P < 0.05; **P < 0.01; ***P < 0.001.

To further assess activation of apoptotic pathways, we examined the levels of additional pro-apoptotic proteins. A slight activation of caspase-8 (membrane death receptor pathway) was observed following treatment with taxol, but caspase-8 was not affected by FKBP5 overexpression (Fig. 3A). In contrast, while caspase-9 (mitochondrial apoptosis pathway) was activated by taxol in a dose-dependent manner, ectopic expression of FKBP5 reduced activation of both caspase-3 and caspase-9 (Fig. 3A).

While release of cytochrome c from mitochondria was induced by taxol, cytosolic cytochrome c levels were reduced by FKBP5 overexpression (Fig. 3B), and the reduction was significant at high dose of taxol (Fig. 3C). Reduced mitochondrial Bax was also observed in FKBP5-overexpressing cells (Fig. 3B and D). These results suggest that the intrinsic, mitochondrial apoptosis pathway is regulated by FKBP5 in ovarian cancer cells.

### FKBP5 forms a protein complex with inactivated AR

**Figure 4:**
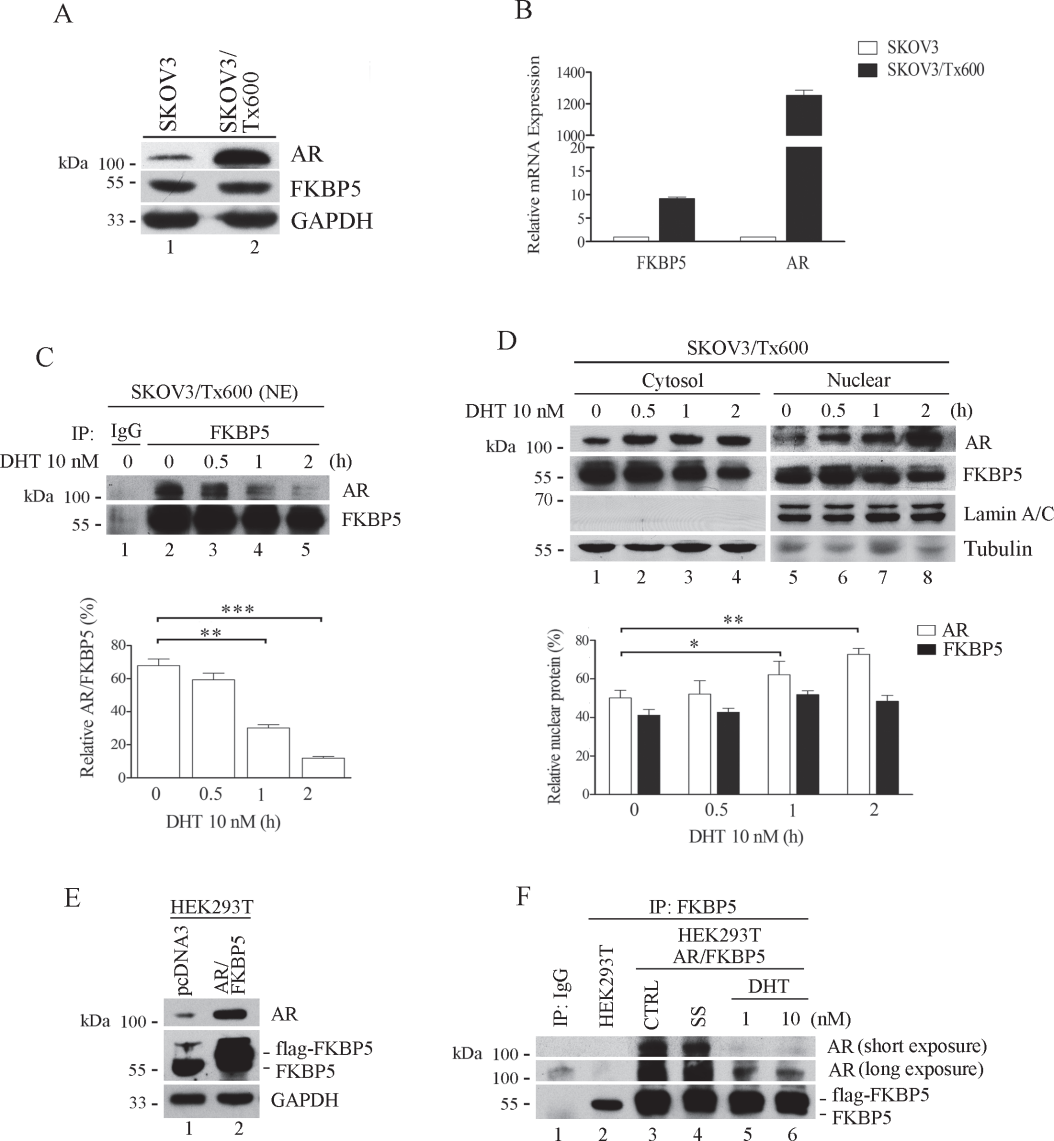
Association of FKBP5 with inactivated AR **(A)** Protein level of FKBP5 and AR in txr and parental cells. **(B)** Upregulated FKBP5 and AR mRNA in txr cells. mRNA level was determined by qPCR. **(C)** Reduced interaction between FKBP5 and activated AR in txr cells. Nuclear AR was detected following AR activation by DHT for the indicated time period. 500 μg of cell extracts were subjected to immunoprecipitation with antibody against FKBP5 or IgG as control. **(D)** While nuclear AR increased, FKBP5 remained unchanged in DHT-treated txr cells. Data of (C) and (D) are expressed as mean values ± SD for experiments performed in triplicate. **(E)** Accumulation of AR protein following FKBP5 overexpression in HEK293T cells. HEK293T cells were transfected with AR and FKBP5 plasmids for 48 h before analysis. **(F)** Reduced association of FKBP5 with activated AR in HEK293T cells. The experiments were performed as in (C), except for cell lines and ectopic expression of FKBP5 and AR (lanes 3–6).

Since FKBP5 represents a scaffold protein and the AR is upregulated in tumors and chemoresistant cancer cells [28, 29], we examined the possibility that these proteins may interact. Although the AR was upregulated in txr ovarian cells (SKOV3/Tx600) compared to parental cells, FKBP5 protein levels were similar in txr and parental cells (Fig. 4A). Nevertheless, FKBP5 mRNA was moderately upregulated in txr cells (Fig. 4B). Consistent with the increased protein level described above, AR mRNA was upregulated more than 1,000-fold in txr cells (Fig. 4B).

Co-immunoprecipitation (co-IP) experiments revealed that the activated AR induced by dihydrotestosterone (DHT) showed reduced interaction with FKBP5

(Fig. 4C). While the level of AR increased following ectopic expression of FKBP5, the level of FKBP5 that precipitated with AR gradually decreased in response to DHT treatment, suggesting that FKBP5 preferentially interacted with inactivated AR.

Activation of AR by DHT was confirmed by increased nuclear translocation of the protein (Fig. 4D, lanes 5–8). In this case, FKBP5 remained in the cytosol and nucleus. Ectopic expression of FKBP5 (flag-FKBP5) induced AR expression in non-tumor HEK293T cells (Fig. 4E). As seen in SKOV3 cells, FKBP5 interacted less efficiently with DHT-activated AR as revealed by co-IP (Fig. 4F, compare lanes 3 and 4 with 5 and 6). These results indicate that the interaction between the two proteins is highly affected by AR's conformation.

### Stabilization of both FKBP5 and AR in txr cells

**Figure 5:**
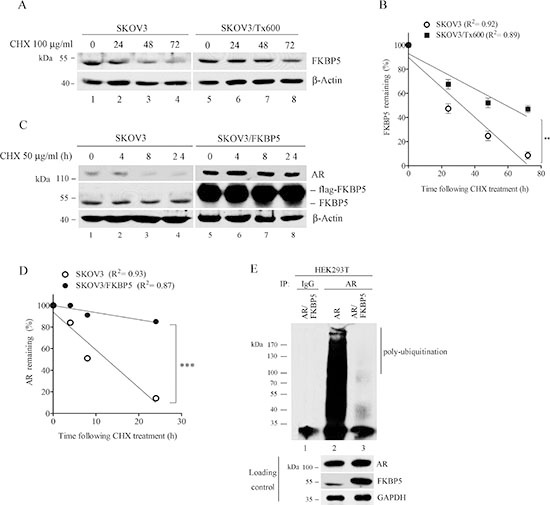
Stable FKBP5 protein in txr cells and stabilization of AR by FKBP5 **(A)** (A, B) Stable protein FKBP5 in txr cells. **(B)** shows quantified data from (A). **(C, D)** Stabilization of AR by FKBP5 overexpression in ovarian cancer cells. (D) shows quantified data from (C). **(E)** Reduced ubiquitination of AR by FKBP5 overexpression. HEK293T cells were overexpressed with FKBP5 and AR for 48 h. MG132 was used to stop proteasome activity for 6 h before immunoprecipitation of the cell extracts with AR antibody or IgG control for detection of the ubiquitinated form.

To assess the stability of FKBP5, we performed experiments using the translation elongation inhibitor cycloheximide (CHX). Following CHX treatment, FKBP5 was stabilized in txr cells (Fig. 5A, compare lanes 1–4 with lanes 5–8). Statistical analysis indicated that the stabilization was significant (Fig. 5B; *P* < 0.01). Ectopic expression of FKBP5 also stabilized AR protein level in parental SKOV3 cells (Fig. 5C and D; *P* < 0.001).

Since protein stability is mostly regulated by ubiquitin-mediated proteasome degradation, we also analyzed the ubiquitination of AR in FKBP5-overexpressing HEK293T cells using treatment with the proteasome inhibitor MG132 [30]. While HEK293T cells displayed highly ubiquitinated AR, the level of this ubiquitinated protein was almost completely eliminated in FKBP5-overexpressing cells (Fig. 5E). These results suggest that FKBP5-induced stabilization of AR may occur through a reduced ubiquitination of the protein. We noted that ectopic expression of FKBP5 resulted in moderate increase of AR in SKOV3 cells, whereas no significant increase of AR was detected in HEK293T cells, suggesting the possibility that higher survival signals may be present in cancer cells.

### FKBP5 is critical for controlling AR expression and cell viability

**Figure 6:**
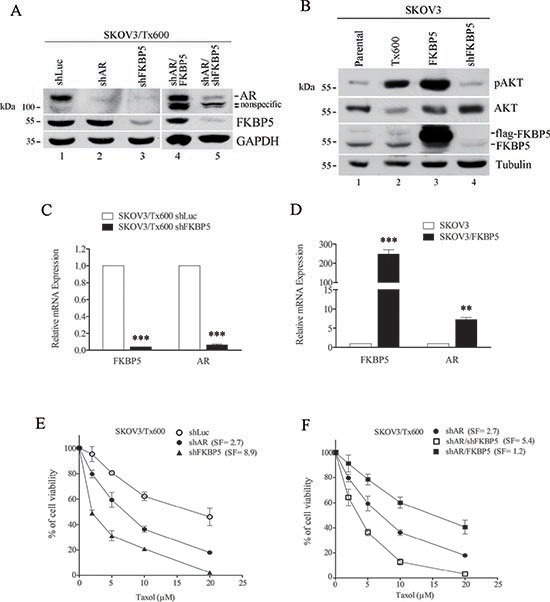
FKBP5-regulated AR levels are associated with taxol sensitivity **(A)** Ectopic expression of FKBP5 rescues AR protein levels in txr cells. **(B)** Inhibition of Akt phosphorylation by FKBP5 silencing. **(C)** Downregulation of AR mRNA by FKBP5 silencing. **(D)** Upregulation of AR mRNA by FKBP5 overexpression. mRNA levels in (C) and (D) were determined by qPCR. **(E)** Reduced cell viability of txr cells following silencing of FKBP5 or AR. **(F)** Rescue of AR-silencing-induced cell death by FKBP5 overexpression. Further reduction of AR-silencing-induced cell viability by silencing FKBP5 is also shown. Modification of cell viability in (E) and (F) are indicated. SF, sensitization factor was calculated from the ratios of IC_50_.

To assess the role of FKBP5 in AR expression and cell response to taxol, we monitored cell viability following silencing or ectopic expression of FKBP5. First, silencing of FKBP5 considerably reduced AR protein expression in txr cells (Fig. 6A). In contrast, ectopic expression of FKBP5 rescued AR expression back to control levels (Fig. 6A, compare lanes 1 and 4).

It is known that high levels of FKBP51 lead to reduced Akt phosphorylation/activity and increased chemosensitivity, whereas low levels of FKBP51 lead to increased Akt phosphorylation/activity and chemoresistance of cancer cells of the prostate, breast, and pancreas [24, 25]. On the other hand, AR stability/activity is regulated by Akt-mediated phosphorylation depending on cell context [31–33]. To examine the possible link between Akt and FKBP5 in ovarian cancer cells, we examined the levels of Akt kinase and found significant inhibition of Akt phosphorylation or activation (pAkt) following FKBP5 silencing (Fig. 6B). These results suggest that FKBP5 positively regulates pAkt and AR expression post-translationally in our system. Similarly, AR mRNA levels were effectively suppressed by FKBP5 silencing (Fig. 6C). In contrast, AR mRNA levels were considerably induced by ectopic expression of FKBP5 (Fig. 6D).

To determine the significance of this alteration, we assessed cell viability following silencing of AR or FKBP5. Silencing of AR produced a 2.7-fold sensitization to taxol, whereas silencing of FKBP5 sensitized the cells by 8.9-fold (Fig. 6E). These results were not due to difference in silencing efficiency (Fig. 6A). Furthermore, silencing of both AR and FKBP5 caused a 5.4-fold sensitization to taxol, whereas ectopic expression of FKBP5 in shAR-expressing cells considerably reduced sensitization, producing a SF of 1.2 (Fig. 6F). These results suggest that FKBP5-regulated txr cell response may occurs through AR expression. However, shFKBP5 induced-sensitization to taxol is remarkably higher than the sensitizing effect induced by shAR. In addition, the sensitization effect of double FKBP5/AR silencing is higher than that of single AR silencing (Fig. 6E). These results suggest that further mechanisms, in addition to AR upregulation, may be involved in FKBP5-regulated taxol resistance.

### FKBP5 regulates a panel of genes associated with txr

**Figure 7:**
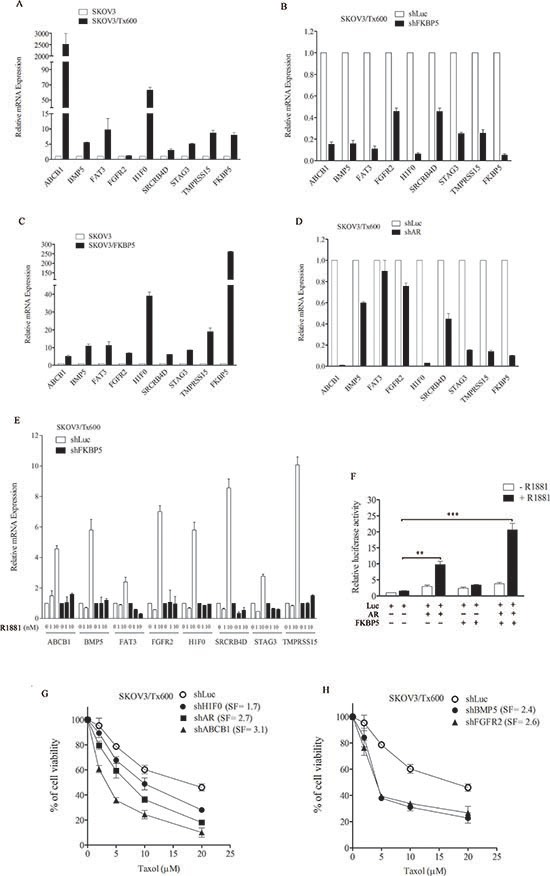
Co-regulation of txr genes by FKBP5 and AR **(A)** Upregulation of representative txr genes in txr cells. **(B)** Downregulation of txr genes by FKBP5 silencing. **(C)** Upregulation of txr genes by FKBP5 overexpression. **(D)** Downregulation of txr genes by AR silencing. **(E)** Upregulation of txr genes following activation of AR by R1881. Note that all txr genes that were upregulated by R1881 were suppressed by FKBP5 silencing in txr cells. **(F)** Further induction of AR-inducible promoter activity by FKBP overexpression in ovarian cells. **(G)** Reduction of cell viability by silencing of ABCB1 or H1F0 in txr cells. The sensitization effect of silencing AR was included for comparison. **(H)** Reduction of cell viability by silencing BMP5 or FGFR2 in txr cells. The symbols are same as for Fig. 6.

Transcriptomic profiling revealed over 100 genes that were upregulated at least 10 fold in txr cells, including ABCB1, BMP5, FAT3, FGFR2, H1F0, SRCRB4D, STAG3, and TMPRSS15. Gene analysis using the MetaCore software revealed that 30 of these genes form an interaction network involving the AR. Ten of these AR-regulated genes (which included two genes that were overexpressed less than 10 fold) and 13 genes that were not regulated by AR were selected and their upregulation was confirmed by qPCR (Table 1). Eight AR-regulated genes were upregulated following ectopic expression of FKBP5 (Table 1; also see Fig. 7). None of the genes that were not regulated by AR was regulated by FKBP5, suggesting that a sub-group of txr genes may be regulated by both FKBP5 and AR. qPCR analysis confirmed the upregulation of these genes (Fig. 7A). FKBP5 silencing downregulated the eight txr gene candidates (Fig. 7B). In contrast, ectopic expression of FKBP5 upregulated these genes by 5 to 40 fold (Fig. 7C).

**Table 1:** Expression levels of upregulated genes in taxol resistant SKOV3/Tx600 cells

Symbol NCBI (NM_ID)	Function	Microarray	Q-PCR	Regulated by FKBP5
		SKOV3/Tx600/SKOV3	SKOV3/Tx600/SKOV3	
*Regulated by AR*				
H1F0 (NM_005318)	nucleosome structure	31.00 ± 1.66**	63.17 ± 8.97***	+
TMPRSS15 (NM_002772)	scavenger receptor and serine-type endopeptidase	30.63 ± 1.09***	8.75 ± 1.21**	+
STAG3 (NM_001282718)	meiosis specific component of cohesin complex	25.30 ± 0.99***	5.11 ± 0.89*	+
FAT3 (NM_001008781)	calcium ion binding	18.70 ± 0.37***	9.78 ± 1.29*	+
BMP5 (NM_021073)	cytokine activity	15.30 ± 0.02***	5.58 ± 0.86*	+
SRCRB4D (NM_080744)	scavenger receptor activity	14.70 ± 0.31***	3.03 ± 0.12	+
FGFR2 (NM_022970)	cell-surface receptor	13.50 ± 0.43***	6.19 ± 0.94*	+
ABCB1 (NM_000927)	transporter	11.00 ± 2.13*	2519.14 ± 394.88**	+
ABCB6 (NM_005689)	transporter	8.09 ± 0.12***	5.90 ± 0.64*	–
ABCG2 (NM_004827)	transporter	4.70 ± 2.15*	17.96 ± 0.49**	–
*Unregulated by AR*				
CCL2 (NM_002982)	chemotactic factor	307.57 ± 4.21**	737.6 ± 42.34***	–
CDH19 (NM_001271028)	calcium-dependent cell adhesion protein	76.37 ± 2.31***	43.58 ± 8.78**	–
BMP4 (NM_130851)	heparin binding and cytokine activity	37.04 ± 4.21**	100.30 ± 4.71***	–
DMD (NM_004010)	structural constituent of cytoskeleton and calcium ion binding	27.60 ± 0.11***	4.13 ± 0.78*	–
PDLIM2 (NM_021630)	myosin heavy chain binding and muscle alpha-actinin binding	20.30 ± 5.69**	2.59 ± 0.44	–
MT1X (NM_005952)	heavy metals binding	19.50 ± 1.15**	72.83 ± 9.26**	–
MAGEB2 (NM_002364)	enhance ubiquitin ligase activity E3 ubiquitin-protein ligases	18.30 ± 0.80**	3.74 ± 0.76*	–
EDN2 (NM_001956)	hormone activity	18.07 ± 0.17***	72.98 ± 8.66***	–
SETBP1 (NM_015559)	nucleosome assembly	15.60 ± 1.10**	1.96 ± 0.22	–
NELL2 (NM_001145108)	calcium ion binding	12.40 ± 0.13***	33.43 ± 2.19	–
CLDN16 (NM_006580)	magnesium ion transmembrane transporter	11.96 ± 0.29*	269.09 ± 44.78**	–
ZNF702P (NM_024924)	transcriptional regulation	10.65 ± 0.53**	11.08 ± 2.36*	–
CD36 (NM_001001548)	lipid binding and transforming growth factor beta binding	8.15 ± 4.32*	10.63 ± 1.65**	–

* *p* < 0.05

** *p* < 0.01

*** *p* < 0.001

Interestingly, silencing of AR also downregulated the txr gene candidates (Fig. 7D). Furthermore, while these genes were upregulated by activation of AR using the synthetic androgen R1881, all of them were suppressed to near basal level following silencing of FKBP5 (Fig. 7E), supporting the notion that FKBP5 and AR interact and control downstream gene expression.

Using an AR promoter reporter assay (see *Materials and methods*), we observed that AR-driven promoter activity was induced 10-fold by R1881, with a basal level of 2.5-fold. R1881-induced AR promoter activity was further upregulated more than 20-fold by ectopic expression of FKBP5 (Fig. 7F).

To assess the role of the potential txr genes identified, we selected the canonical multidrug gene ABCB1 and a novel H1F0 for cell viability experiments. Silencing of ABCB1 and H1F0 sensitized txr cells to taxol by 3.1 fold and 1.7 fold, respectively (Fig. 7G). Furthermore, silencing of BMP5 and FGFR2 resulted in 2.4-fold and 2.6-fold sensitization of txr cells to taxol, respectively (Fig. 7H). Further experiments showed that individual silencing of the other four FKBP5/AR-regulated genes sensitized txr cells to taxol (Fig. S2).

### Silencing FKBP5 sensitizes mouse tumor xenograft to taxol treatment

To assess whether the modulation effects of FKBP5 on txr exists *in vivo*, we used a mouse tumor xenograft model. SKOV3 cells were implanted into severe combined immunodeficiency (SCID) mice by a two step transplantation process due to difficulties in colonizing the animals with this cell line. Prepared SKOV3 cells were expanded in cell culture for silencing FKBP5 or control shLuc. When SKOV3 cells of both groups were grown subcutaneously to the same volume, animals were injected with saline (untreated) or with taxol, and followed until tumors reached ~1 cm in diameter.

**Figure 8:**
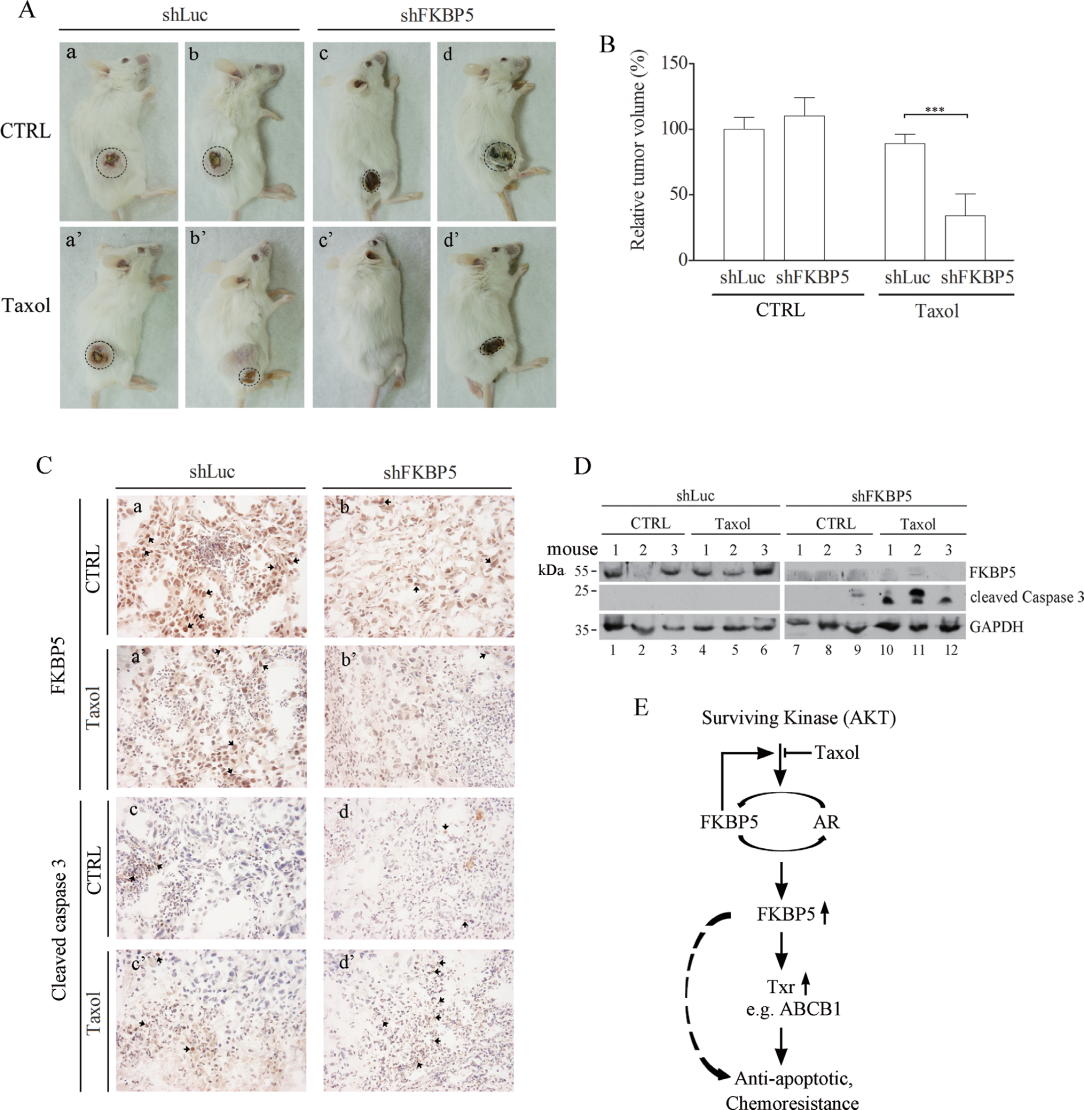
Sensitization to taxol by combined shFKBP5/taxol treatment in SCID mice xenografts **(A)** Representative SCID mice with tumors 80 days post-inoculation. SCID mice 50 days post-inoculation were left untreated or were repeatedly treated with taxol for 30 days. **(B)** Reduction in tumor volume of mice xenografts following repeated taxol injections of the indicated cells. The data were reported as mean values ± SD of six mice. **(C)** Detection of FKBP5 in shLuc and shFKBP5 mouse tissues by immunohistochemistry. (a, a') FKBP5 detection of shLuc mouse tissues with or without taxol treatment. (b, b') FKBP5 protein levels of shFKBP5 mouse tissues with or without taxol treatment. (c, c') Apoptosis (cleaved caspase-3) in shLuc mouse tissues. (d, d') Apoptotic (cleaved caspase-3) detection of shFKBP5 mouse tissues. FKBP5 or apoptotic staining (brown) in tumor tissues is indicated with arrows. Note that more apoptotic cells were detected in shFKBP5 tumor than control shLuc following taxol treatment. The size reference (25 μm) was indicated. **(D)** Taxol-induced caspase activation is dramatically enhanced following silencing of FKBP5 as revealed by Western blotting of mouse tumors. **(E)** Working model of FKBP5 and related signal pathway implicated in txr in ovarian cancer cells. While cells untreated with taxol maintain protective Akt-dependent FKBP5/AR protein expression for growth, taxol-treated cells show reduced AR levels. The non-activated AR forms a complex with FKBP5, leading to upregulation of genes involved in txr. FKBP5 may also activate other genes in the nucleus. Both pathways eventually lead to anti-apoptotic effects or chemoresistance.

Representative animals showed reduced tumor size following silencing of FKBP5 and treatment with taxol (Fig. 8A). We noted that the tumors were covered by a dried tissue that may represent shrinked tumor cells, which were present even without any treatment. While tumor volume was the same between untreated shLuc control and shFKBP5 groups, it was significantly reduced in the FKBP5 silencing group following taxol treatment (Fig. 8B; *P* < 0.001).

To assess the status of these tumors, FKBP5 expression and apoptotic cells were examined. Immunohistochemistry staining showed that FKBP5 expression was reduced in shFKBP5 tumors treated or not with taxol (Fig. 8C, panels a, a', b, and b'; indicated with arrows). Apoptotic cells revealed by activated caspase-3 expression were enhanced following silencing of FKBP5 in taxol-treated tumors (Fig. 8C, compare shLuc panels c and c' and shFKBP5 panels d and d'; indicated with arrows). Furthermore, taxol-induced caspase activation was dramatically enhanced following silencing of FKBP5 as revealed by Western blotting performed on mouse tumors (Fig. 8D).

## DISCUSSION

In this study, we found that FKBP5 represents a powerful regulator or driver of gene expression involved in txr. By comparing genome-wide expression of genes in parental and txr ovarian carcinoma cells, we identified over 100 genes that were overexpressed more than 10-fold in txr cells. We found that eight txr genes were upregulated by FKBP5/AR. Surprisingly, FKBP5 was initially upregulated over 100-fold in txr cells but its expression was reduced following prolonged culture as measured by DNA microarray and qPCR. FKBP5 nonetheless upregulated a sub-group of txr genes in resistant cells. Our results confirm that FKBP5 acts as a scaffold protein [13] that recruits AR and regulates gene expression. Amplification of FKBP5 expression also subsequently upregulates target txr genes such as ABCB1, H1F0, BMP5, and FGFR. However, our current data did not show whether FKBP5 can directly function to regulate taxol response in ovarian cancer cells. Most of the txr genes identified are novel associations worthy of further studies to verify their role in regulation of txr. While the txr genes are highly upregulated and readily detected by transcriptome profiling, FKBP5 may have been overlooked due to its transient upregulation at the transcriptional level. Accordingly, we uncover a potentially hidden marker that plays a role in the development and/or maintenance of txr (a diagram of the pathway involved in txr is shown in Fig. 8E).

We noted that FKBP5 protein levels were more stable in txr cells than in parental cells. Cells in which FKBP5 was overexpressed displayed a considerable stabilization of the AR protein, which is a versatile protein capable of reprogramming genomic activity in prostate cancer and probably also in other cancers [28]. Our results indicate that a normal level of FKBP5 is able to stabilize AR or lead to its accumulation in ovarian cancer as well as in non-cancer HEK293T cells. Interestingly, FKBP5 appears not to form a complex with activated AR following stimulation with DHT in our cell system. This phenomenon may be explained by the notion that FKBP5 acts as a scaffold protein that recruits other components in an appropriate conformation through TPR domains [17]. Txr genes may be upregulated through AR activity during the development of txr, as long as FKBP5 provides the required factors [28]. This scenario is highly possible since AR, through phosphorylation [34] or ubiquitination/deubiquitination [35, 36] by other proteins, may be structurally modified and this process may regulate the transcription of target genes. In addition to interacting with AR, FKBP5 also interacts with other hormone-regulated receptors such as PR and GR to regulate target genes [17]. However, a network construction using MetaCore and the highly upregulated genes or potential txr genes identified here did not reveal a significant association with PR or GR (data not shown). These results suggest that FKBP5 preferentially recruits AR and other protein components for upregulation of txr genes in the process of txr.

Another interesting finding reported here is that the regulatory role of FKBP5 in chemoresistance appears to be specific for drugs that impair mitotic functions. This observation is supported by the lack of regulation in cells treated with cisplatin, which mainly causes DNA damage. Highly-overexpressed genes in cisplatin-resistant cervical cancer cells have been identified recently, and these genes have been found to be involved in resistance to cisplatin [37]. Of note, we observed no overlapping between the genes upregulated by cisplatin or taxol. The transcriptomic profiling performed here identified genes that may be involved in response to other mitotoxins as the txr cells were also resistant to the mitotic inhibitor vincristine [38]. Since ABCB1 has been shown to represent an important factor for multiple drug resistance [3], the finding that the FKBP5/AR/ABCB1 axis plays a role in txr in ovarian cancer cells supports the usefulness of our strategy.

Our results, although not consistent with those of Pei et al, who showed that FKBP51 reduces pAkt levels [25], are, instead, in line with those of Fabian AK et al, [39] who, using mutational and pharmacological studies, showed that FKBP inhibitors are unlikely to inhibit the Akt-FKBP-PHLPP network.

Additional factors that interact with FKBP5 may provide an additional layer of regulation and this regulation may be cell-type dependent. Our findings strongly suggest that regulation of txr genes by FKBP5 is specific to the drug used. Identification of unique txr genes may provide important markers for the development of new cancer treatments based on single or multiple drug(s).

## MATERIALS AND METHODS

### Cell lines and reagents

SKOV3 cells (American Type Culture Collection, Rockville, MD, USA) were grown as monolayers in a 1:1 mixture of DMEM/nutrient F-12 Ham (Life Technologies, Grand Island, NY, USA) supplemented with 1% (w/v) penicillin/streptomycin and 10% (v/v) fetal bovine serum (FBS) at 37°C in a humidified atmosphere containing 5% CO_2_. The chemotherapeutic drugs used included cisplatin, taxol (paclitaxel), and vincristine (Bristol-Myers Squibb, New York, NY, USA). MG132 and cycloheximide were purchased from Calbiochem/Merck (Darmstadt, Germany). The other chemicals were purchased from Sigma-Aldrich (St. Louis, MO, USA). All reagents were used according to the instructions provided by the supplier.

### Establishment of cell lines with acquired resistance to taxol

Taxol-resistant ovarian cancer cells were obtained from the parental, drug-sensitive, ovarian cancer cell line SKOV3 by administrating taxol in a conventional dose-escalation manner. The concentration of taxol was increased stepwise, starting at 10 nM and finishing at 600 nM. Parental SKOV3 cells were first exposed to 10 nM taxol for two months followed by exposure to stepwise double concentrations of taxol for a further two months of treatment. Chemoresistant cell lines were maintained in selective medium containing the taxol concentration used for selection of resistance. The cells were cultured in taxol-free medium for one week before further studies. Episodic determinations of inhibitory concentration 50% (IC_50_) values confirmed that taxol-resistant phenotypes were stable for at least two months in drug-free medium.

### Cell viability

Cell growth inhibition were determined using the *in vitro* MTT [3-(4,5-dimethylthiazol-2-yl)-2, 5-diphenyl-2*H*-tetrazolium bromide] colorimetric method as previously described [40]. IC_50_ of cell viability was defined as the levels that caused 50% reduction in the cell viability treatment versus the DMSO control. Resistance factors (RF) or sensitization factors (SF) were calculated based on IC_50_ ratios to express the levels of resistance or sensitization.

### Real time q-PCR analysis

Total RNA was extracted with the Trizol reagent (Life Technologies) as previously described [41]. RNA concentrations were assessed using spectrophotometry and only the samples with an A_260_/A_280_ ratio between 1.9 and 2.2 were used. Real-time q-PCR was performed on total RNA as before [41]. The primers used were as follows: FKBP5 forward, TTTGACTGCAGAGATGTGGC, reverse CCTGCCTCTCCAAAACCATA; AR forward, CGGAAGCTGAAGAAACTTGG, reverse, ATGGGCTGACATTCATAGCC; ABCB1 forward, GTTCAAACTTCTGCTCCTGA, reverse, CCCATCATTGCAATAGCAGG; BMP5 forward, GGCAGAAGAGACCAGAGGGGCA, reverse, TGGGTGGTCAGAGGAGTCGTCC; FAT3 forward, CGGCCGCAACGTCTACCAGG, reverse, TCAGGATGCGGGGCGACTCA; FGFR2 forward, GAGTTGCTCCCCGCAACCCC, reverse, CCGCGACCTGTGTTGTCCCC; H1F0 forward, TGGCTGCCACGCCCAAGAAA, reverse, TCTTGCCGGCCCTCTTGGCA; SRCRB4D forward, TGGGGGTGGAGGTTGGGAGATG, reverse, TGGCCAGTGGCAGGAGGAGAA; STAG3 forward, CGGAAACAGTCAGAGCCACCAGC, reverse, ACTGCATGTCACTTTTGGCGGC; TMPRSS15 forward, TATGGCGGCCGACTGCTCTG, reverse, TACACGCAGTGTGCGGCGG.

### DNA microarray

Fluorescent RNA targets were prepared from 2.5 μg total RNA isolated from parental SKOV3 and taxol-resistant derivative cells using the OneArray Amino Allyl aRNA Amplification Kit (Phalanx Biotech Group, Hsinchu, Taiwan) and Cy5 dyes (GE Healthcare, Little Chalfont, UK). Fluorescent targets were hybridized to the Human Whole Genome OneArray v5.1 which contains 30,275 DNA oligonucleotide probes (HOA5.1, Phalanx Biotech Group, Hsinchu, Taiwan) with Phalanx hybridization buffer and system. After 16 hrs of hybridization at 50°C, non-specific binding was washed away using three different washing steps (wash 1, 42°C for 5 mins; wash 2, 42°C for 5 mins and 25°C for 5 mins; wash 3, rinse 20 times). The slides were dried by centrifugation and scanned using an Axon 4000B scanner (Molecular Devices, Sunnyvale, CA, USA). Cy5 fluorescent intensity of each spot was analyzed using the GenePix Pro 4.1.1.44 software (Molecular Devices). Repeat experimental data were tested with the Pearson correlation coefficient calculation to check reproducibility (R value > 0.975). Signal intensity of each spot was transferred to the Rosetta Resolver System (Rosetta Biosoftware, Seattle, WA, USA) for data analysis. The error model of the Rosetta Resolver System removed both systematic and random errors. Spots that passed the selection criteria were normalized by the median scaling normalization method. Normalized intensities were transformed to gene expression log 2 ratios between control and treatment groups using the Rosetta Resolver error model adjustment. Fold change values were calculated from adjusted log 2 ratios and were used to select differential expression genes. The primary data was deposited into the GEO database (GSE58878 containing subseries GSE58840, GSE58877, and GSE60335). Gene network analysis was performed using the MetaCore software (Thomson Reuters, Philadelphia, PA, USA).

### Plasmids, transfection, cell extracts, and immunoblot analysis

The pSG5-AR AR-expressing plasmid used in these experiments was provided by Dr Hsiu-Ming Shih, Academia Sinica, Taiwan). Preparation of cell samples and procedures of Western blotting were performed as described before [37]. Fifty μg of protein sample was separated on a 10% sodium dedecyl sulfate-polyacrylamide gel [42] and electro-blotted onto polyvinylidene difluoride (PVDF) membranes (Millipore, Bedford, MA, USA). After electroblotting, the membranes were incubated in 5% non-fat dry milk in Tris-buffered saline/Tween 20 (0.1 M Trizma base, 0.15 M NaCl, 0.05% Tween 20, pH 7.4) for blocking and with the primary antibody raised against the following proteins: cleaved caspase-3, Akt, phosphor-Akt (Cell Signaling Technology, Danvers, MA, USA), AR, GAPDH (FL-335), FKBP5 (H-100), β-actin, Bax (N-20), PARP (H-250) (Santa Cruz Biotechnology, Santa Cruz, CA, USA). Membranes were incubated with secondary antibodies: goat anti-mouse or goat anti-rabbit horseradish peroxidase (Amersham, Buckinghamshire, UK). Resulting protein signals were visualized by enhanced chemiluminescence based on the specifications of the supplier (Pierce, Rockford, IL, USA). Protein band intensity was determined by scanning X-ray films through a scanning densitometer (GS 300, Hoefer, Holliston, MA, USA).

### shRNA-mediated gene knockdown

Knockdown of candidate genes was performed using pLKO.1 plasmids expressing shRNA purchased from the National RNAi Core Facility (Academia Sinica, Taipei, Taiwan) as described before [37]. The shRNA clone identification numbers and target sequence were as follows: FKBP5, TRCN0000000237, CCCTCGAATGCAACTCTCTTT; AR, TRCN0000003715, CCTGCTAATCAAGTCACACAT; BMP5, TRCN0000371431, ATGCCACCAACCACGCTATAG; FGFR2, TRCN0000219680, TGGAGTACTCCTATGACATTA; ABCB1, TRCN0000059684, GCAGCAATTAGAACTGTGATT. Luciferase shRNA (TRCN0000072244, ATCACAGAATCGTCGTATGCA) was used as a negative control.

### Luciferase AR promoter analysis

The AR-responsive ARE-directed luciferase reporter (pMMTV-luc, provided by Dr. Chawnshang Chang) and AR expression plasmid (pSG5-AR, provided by Dr Hsiu-Ming Shih, Academia Sinica, Taiwan) were used in this study. Healthy, growing cells were co-transfected with or without pSG5-AR and pMMTV-luc gene constructs with Lipofectamine following the procedures provided by the supplier (Invitrogen). pRL-GFP transfection was used for normalization of transfection efficiency. Sixteen h after transfection, the cells were treated with either solvent (ethanol) or 10 nM R1881 for 24 h, prior to reporter analysis. Luciferase activity was measured using the Luciferase Assay System (Promega).

### Ovarian tumor xenografts in SCID mice

Animal handling and experimental procedures were approved by the Animal Experimental Ethics Committee of Chang Gung University. Female SCID mice (CB17/Icr-Prkdc^scid^/Cr1Nar1) of six weeks of age were purchased from the National Laboratory Animal Center (Taipei, Taiwan). Tumors were produced by subcutaneous injection of 1 × 10^7^ SKOV3-shLuc cells and 1 × 10^7^ SKOV3-shFKBP5 cells into six SCID mice each. Tumor size was measured as described before [43].

### Statistical analysis

Data were reported as mean values ± standard deviation (SD). Three independent experiments were performed unless indicated otherwise. Statistical significance (*P* value) was calculated with a two-tailed Student's *t* test for single comparison.

## SUPPLEMENTARY FIGURES



## Abbreviations

Akt: Ak strain thymoma
DMSO: dimethyl sulfoxide
FBS: fetal bovine serum
FKBP5: FK506-binding protein 5
GAPDH: glyceraldehyde-3-phosphate dehydrogenase
MDR: multiple drug resistance
MTT: 3-(4,5-dimethylthiazol-2yl)-2,5-diphenylterazolium bromide
NF-κB: nuclear factor-κB
PARP: poly-ADP ribose polymerase
PBS: phosphate-buffered saline
PCR: polymerase chain reaction
PVDF: polyvinylidene fluoride
q-PCR: quantitative polymerase chain reaction
SDS-PAGE: sodium dodecyl sulfate-polyacrylamide gel electrophoresis
shRNA: short-hairpin RNA
TKI: tyrosine kinase inhibitor
