# Supplementary material for: Transcriptomic profiling of taxol-resistant ovarian cancer cells identifies FKBP5 and the androgen receptor as critical markers of chemotherapeutic response
Source: Oncotarget. 2014 Nov 17;5(23):11939–56. doi: 10.18632/oncotarget.2654 (PMC4322968; doi:10.18632/oncotarget.2654)
Supplement: Supplementary file 1 [file oncotarget-05-11939-s001.pdf]

SUPPLEMENTARY FIGURES

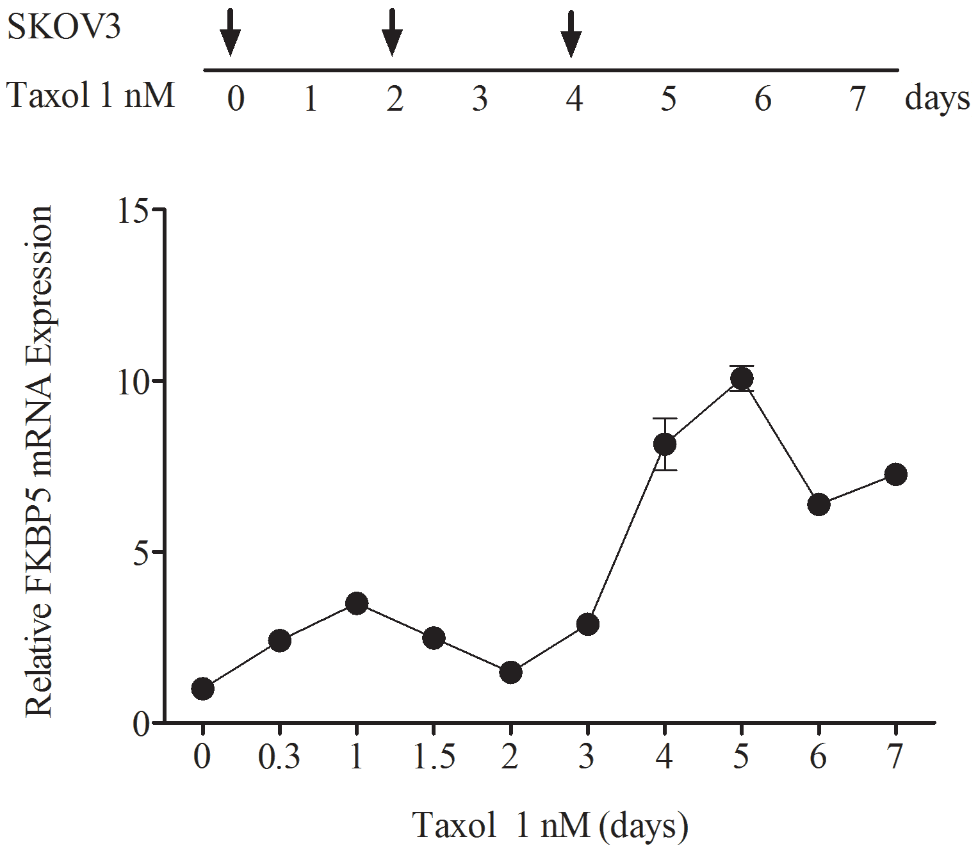

Supplementary Figure S1: mRNA levels of FKBP5 in early response to taxol treatment in SKOV3 cells. The level of FKBP5 mRNA following taxol treatment was quantified by qPCR and is presented relative to day 0. The average values of each time point were initially normalized against 18S rRNA level, which was used as internal control.

SKOV3/Tx600

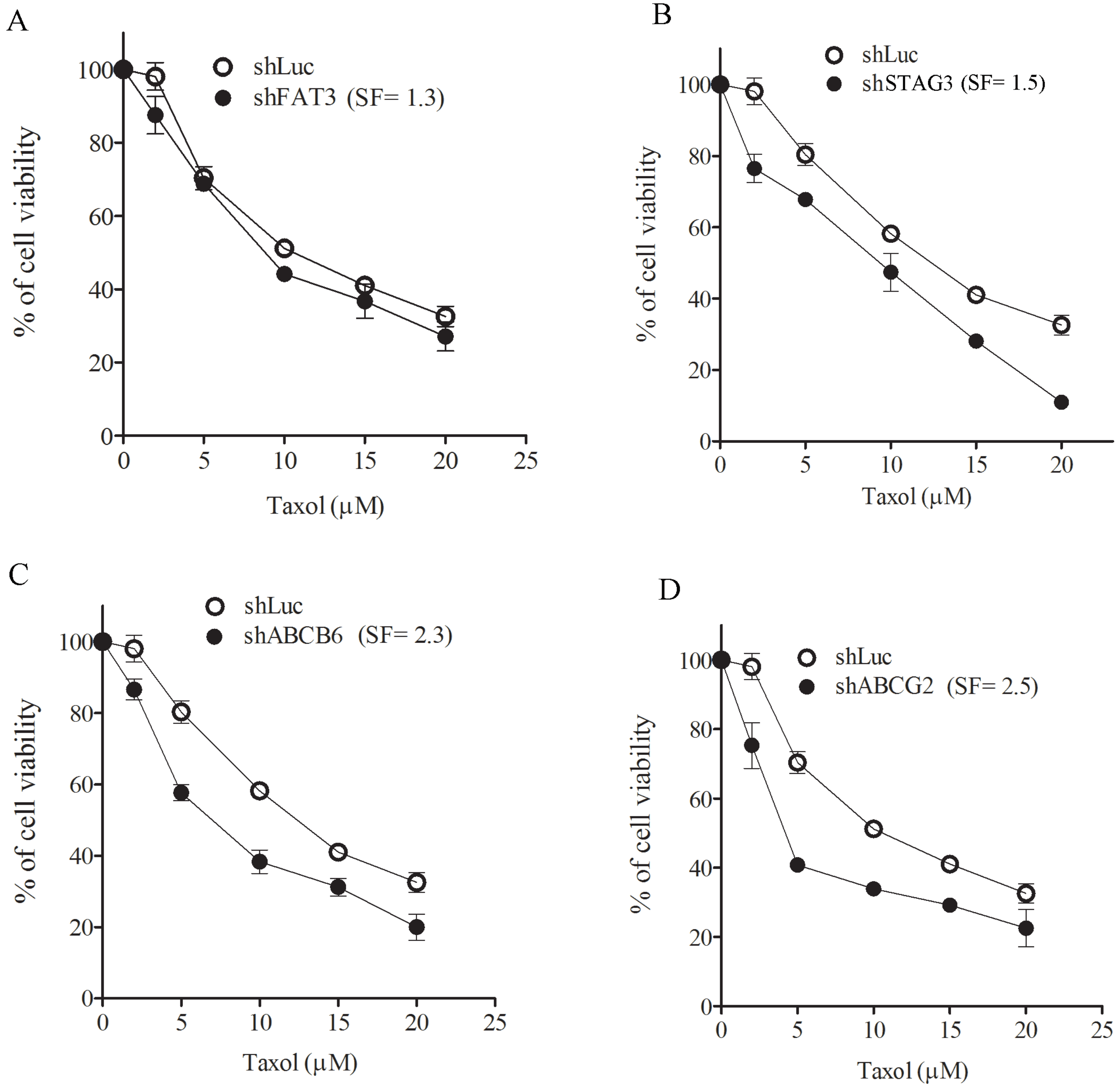

Supplementary Figure S2: Silencing of FKBP5-regulated genes sensitizes ovarian cancer cells to taxol. Cell sensitization to taxol following silencing of FAT3 **(A)** STAG3 **(B)** ABCB6 **(C)** or ABCG2 **(D)**. Cell viability was assessed by the MTT assay. Modification of cell viability is indicated by a sensitization factor (SF), calculated from the ratios of IC_{50}. The results are expressed as mean values ± SD for experiments performed in triplicate.
